# Prevalence of Dry Eye Disease and Its Risk Factors Among the General Population of Saudi Arabia: A Cross-Sectional Survey

**DOI:** 10.7759/cureus.32552

**Published:** 2022-12-15

**Authors:** Saif K Dossari, Ahmed Z Alkhars, AlReem A Albaqshi, Hajar M AlHajri, Zainab A Alabdullah, Zuhair A Almuhnna, Bureer A Almuhanna, Mohammed A Aljanobi

**Affiliations:** 1 Ophthalmology, King Faisal University, Hofuf, SAU; 2 General Physician, AlJaber Eye and ENT Hospital, Hofuf, SAU; 3 General Physician, Primary Health Care Corporation, Hofuf, SAU; 4 Dentistry, Primary Health Care Corporation, Hofuf, SAU

**Keywords:** epidemiology, prevalence, cornea, lacrimal system, dry eye disease

## Abstract

Background: Dry eye disease is a multifactorial chronic disorder of the ocular surface, which leads to symptoms of discomfort and distress. Dry eye disease is a global health concern and is one of the most frequent ocular diseases encountered in an ophthalmology clinic. The estimated prevalence of dry eye disease in the literature ranged from 7.4% to 93.2%. Saudi Arabia’s population, especially in the eastern province, is at great risk of developing dry eye disease, however, there is hardly any nationwide study that assesses the prevalence of dry eye disease among the general population and its risk factors.

Aim: The aim is to estimate the prevalence of dry eye disease and determine its risk factors among the general population of Saudi Arabia.

Methods: This study was a cross-sectional study conducted on the general population of Saudi Arabia between September 2022 and November 2022. A convenient sampling technique was deployed for participant recruitment, where a self-administered questionnaire was created and dispersed to the general population all over the country with an invitation to participate in the study. Dry eye disease prevalence was assessed using Ocular Surface Disease Index survey. The Chi-square test was used to test for factors associated with the prevalence of dry eye disease, and undiagnosed dry eye disease. Multivariate logistic regression was also used to determine risk factors for dry eye disease.

Results: A total of 1,381 participants were included in this study. The prevalence of dry eye disease among the general population of Saudi Arabia was observed to be (17.5%). Among the participants (11%) had mild dry eye disease, (4.7%) had moderate dry eye disease, and (1.7%) had a severe dry eye disease. Among those observed to have a dry eye disease, (58.09%) were not previously diagnosed. The following factors were observed to be significantly associated with having dry eye disease, being female, having thyroid disease, having systemic lupus erythematosus/rheumatoid arthritis, using antidepressants, using antihistamine/decongestants, using electronic devices for a prolonged time, using contact lenses, having a history of eye surgery, history of conjunctival/eyelid infection, and history corneal abrasions/erosions/ulceration.

Conclusion: This study revealed that the prevalence of dry eye disease among the general population of Saudi Arabia is notably lower than what was observed in other local studies and similar to what was found in some global studies. Adjusted binary multivariate logistic regression revealed that the risk factors were only: being female, using antidepressants, using antihistamines/decongestants, and prolonged use of electronic devices.

## Introduction

Dry eye disease (DED) is a multifactorial chronic disorder of the ocular surface, which leads to symptoms of discomfort and distress [1-4]. Elevated tear evaporation, depressed tear production, and poor quality of tears are characteristic features of DED [1, 4]. Affected individuals usually suffer from visual acuity disturbances, itchiness, burning sensation, fatigue, irritation, and photophobia [4]. Although these patients do not endure dreadful events such as severe visual impairment and blindness, they sustain serious consequences that highly affect their quality of life and interfere with daily activities [1,4]. It was found that these individuals are more prone to ocular infections as well as ocular surface destruction. Moreover, in severe cases, they may experience abrasions and corneal ulcerations [4].

DED is a global health concern. It is one of the most frequent ocular diseases encountered in an ophthalmology clinic [1-4]. The estimated prevalence of DED as reported by Bukhari et al. and Alshamrani et al. ranges from 7.4% to 33.7% [2,3]. This wide variation can possibly be explained by the difference in the characteristics of the investigated population and their geographical location [1-4]. Although there is a diversity of definitions for DED, the definition that is often used to determine dry eye prevalence in population-based studies is the one based on dry eye symptoms other than the objective clinical tests in identifying dry eyes [2,4]. Many tools were developed to screen for the prevalence of DED, among these tools is a validated six-item questionnaire of ocular symptoms related to a dry eye used in previous literature [5], and the validated 12-item questionnaire (Ocular Surface Disease Index [OSDI]) [6].

Saudi Arabia's population, especially in the eastern province, is at a great risk of developing DED, which is due to the environmental and epidemiological risk factors of this region [2]. Like most of the Saudi Arabian regions, the eastern province is known to have one of the hottest desert climates that reach up to 50 degrees in summer, which stands as a great risk factor for DED [2].

Although several studies have been conducted to estimate the epidemiology of dry eye in different countries, the literature is deficient in regard to a nation-wide study that estimates the prevalence of DED among the residents in Saudi Arabia and the risk factors associated with it [2,4]. For that reason, this study aimed to estimate the prevalence and determine the risk factors of DED among residents of Saudi Arabia.

## Materials and methods

Study design and settings

 This study was a cross-sectional study that targeted the general population in Saudi Arabia to screen for the prevalence of DED and determine its risk factors. The study was conducted between September 2022 and November 2022.

Study subjects, inclusion, and exclusion criteria

The study targeted subjects were all the adults living in Saudi Arabia who consented to participate in the study during its period between September and November 2022 and have met the inclusion and exclusion criteria.

The inclusion criteria consisted of being an adult aged 18 years and older, living in Saudi Arabia, and having consented to participate in the study.

The exclusion criteria consisted of being younger than 18 years old, living outside of Saudi Arabia, and not consenting to participate in the study.

Sampling and sample size

The used sampling technique was convenient random sampling where the questionnaire was spread, and the general population of Saudi Arabia was invited to participate through an online link. The sample size was calculated using the formula n = z2pq\d 2. With a confidence level of 95%, an estimated proportion of 50% and a 5% level of precision. The minimum sample size was calculated to be 385. However, more participants and candidates were included to ensure the sufficiency and accuracy of the results.

Data collection

An online survey was created using Google forms for data collection. The online survey was dispersed to the general population in each of the five regions of Saudi Arabia, respectively (central, eastern, northern, western, and southern regions) and people were invited to participate. In order to reach the population from each region of the five regions, and in order to enroll as many participants as possible, two data collectors were recruited from every region. Data were collected through an online self-administered questionnaire where participants first consented to participate in the study before starting to fill out the questionnaire. The questionnaire included five sections, the first section asked about socio-demographical data including age, and gender. The second part asked about past medical history which included asking about diabetes mellitus (DM), thyroid diseases, rheumatoid arthritis, or systemic lupus erythematosus, as well as the history of utilization of antidepressant medications, antihistamine drugs, or decongestants. The third part screened for the presence of other causes of dry eye symptoms which included a history of smoking or getting exposed to fumes on a regular basis, a history of spending prolonged time using electronic devices, and a history of usage of contact lenses. The fourth section navigated the history of ocular diseases like eyelid infections, cataract, glaucoma, corneal ulcers, and previous ocular surgeries. The fifth and last part was the pre-validated OSDI survey to screen for the prevalence of DED.

Study tool and its validation

A survey was constructed and developed by the investigators and presented to specialists in ophthalmology for improvement and approval. The survey was formed in English and then was translated into Arabic for it to be comprehensible for the targeted population. The Arabic version was first examined by three different language experts and the translation was approved after grammatical and linguistic modifications. After that, a pilot study was performed on a small group of people (15 persons) to confirm a uniform understanding of the questions. Part of the questionnaire utilized the pre-validated OSDI survey to screen for the prevalence of DED [6]. In accordance to the interpretation guidelines of the OSDI survey, those who had an OSDI score of (12 and less) were considered to be normal (do not have DED), those who had a score between (13-22) were considered to have mild DED, those who had a score of (23-32) were considered to have a moderate DED, while those who had a score of (33-100) were considered to have severe DED.

Data management and statistical analysis

Data analysis was performed using Statistical Package for the Social Sciences, SPSS 23rd version. Frequency and percentages were used to display categorical variables. Minimum, maximum, mean, and standard deviation were used to present numerical variables Chi-square test was used to test for factors associated with having DED and being undiagnosed with DED. Furthermore, binary logistic regression was utilized to determine the risk factors for having DED. The following factors were included in the logistic regression model (age, gender, diabetes, thyroid disease, systemic lupus erythematosus or rheumatoid arthritis, using antidepressants, using antihistamine or decongestant, being a smoker or being regularly exposed to fumes/smoke, using contact lenses, having cataract, having glaucoma, having a history of eye surgery). Model fitness-of-good was tested using the Omnibus test and Hosmer and Lemeshow test. The level of significant was set at 0.05.

Confidentiality and ethical consideration

Data were managed with the highest level of confidentiality. Privacy was ensured throughout the study steps. Ethical approval was obtained from the ethical board in the deanship of scientific research at King Faisal University, Saudi ArabiaArabia (KFU-REC-2022-NOV-ETHICS354).

## Results

A total of 1,381 participants were included in the study. Table 1 shows the socio-demographic profile of the participants. The minimum age was 18, the maximum was 90, and the mean was 32.53 +/- 12.35. As for the age groups the participants belonged to, 753 (54.5%) were between 18 and 30 years, 395 (28.6%) were between 31 and 45 years, 207 (15%) were between 46 and 60 years, and 26 (1.9%) were 61 years and older. As for gender, 532 (38.5%) of the participants were males, while 849 (61.5%) of the participants were females.

**Table 1:** Socio-demographic profile of the participants (n = 1,381)

Demographic Characteristics	n	%
Age		
Minimum	18	
Maximum	90	
Mean	32.53	
Standard deviation	12.35	
Age		
18-30 years	753	54.50
31-45 years	395	28.60
46-60 years	207	15.00
61 years and older	26	1.90
Gender		
Male	532	38.50
Female	849	61.50

**Figure 1:**
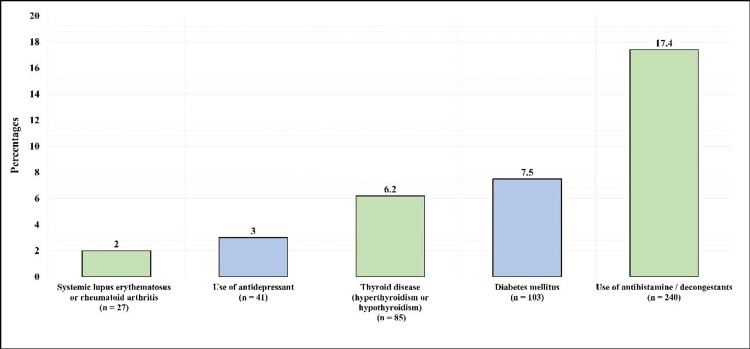
Medical history of the participants

Figure 1 displays the medical history of the participants. Twenty-seven (2%) of the participants reported having systemic lupus erythematosus/rheumatoid arthritis, 41 (3%) reported using antidepressants, 85 (6.2%) reported having thyroid disease (hyperthyroidism/hypothyroidism), 103 (7.5%) reported having DM, and 240 (17.4%) reported using antihistamine/decongestants.

**Figure 2:**
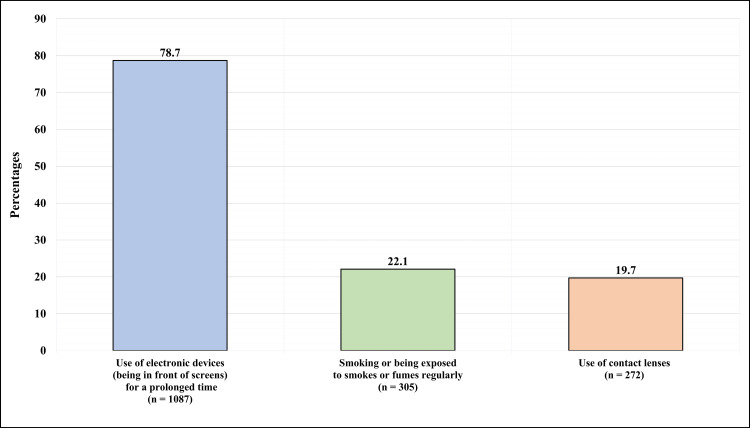
Participants’ attitude/environmental exposure that is associated with dry eye disease

Figure 2 presents the participants’ attitude/environmental exposure that is associated with DED. 1,087 (78.7%) reported using electronic devices (being in front of screens) for a prolonged time, 305 (22.1%) reported smoking or being exposed to smoke or fumes regularly, and 272 (19.7%) reported using contact lenses.

Table 2 demonstrates the ocular history profile of the participants. 395 (28.6%) reported having a history of conjunctival/eyelid infection, 200 (14.48%) reported having a history of eye surgery, 143 (10.35%) reported having a history of corneal abrasions/erosions/ulceration, 26 (1.88%) reported having cataract, and 13 (0.94%) reported having glaucoma. Among the participants, 457 (33.1%) were previously diagnosed with DED. Moreover, 631 (45.7%) reported using eye lubricant drops or gel, while 750 (54.3%) reported they do not.

**Table 2:** Ocular history profile of the participants (n = 1,381)

Question	n	%
Ocular History		
Conjunctival infections/eyelid infections	395	28.60
Eye surgery	200	14.48
Corneal abrasions/erosions/ulceration	143	10.35
Cataract	26	1.88
Glaucoma	13	0.94
Previous Diagnosis of Dry Eye Disease		
Previously diagnosed with dry eye disease	457	33.10
Never diagnosed with dry eye disease	924	66.90
Use of Eye Lubricants Drops or Gel		
Used eye lubricants drops or gel	631	45.70
Never used eye lubricants drops or gel	750	54.30

Table 3 illustrates participants’ responses toward the OSDI survey. The minimum score of the participants was 0, the maximum was 48, and the mean was 6.65 + 8.1.

**Table 3:** Participants’ responses toward the ocular surface disease index survey (n = 1,381)

Question	n	%
Have you experienced any of the following during the last week?		
Q1/ Eyes that are sensitive to light?		
None of the time	803	58.10
Some of the time	386	28.00
Half of the time	60	4.30
Most of the time	95	6.90
All of the time	37	2.70
Q2/ Eyes that feel gritty?		
None of the time	940	68.10
Some of the time	269	19.50
Half of the time	65	4.70
Most of the time	73	5.30
All of the time	34	2.50
Q3/ Painful or sore eyes?		
None of the time	776	56.20
Some of the time	377	27.30
Half of the time	95	6.90
Most of the time	101	7.30
All of the time	32	2.30
Q4/ Blurred vision?		
None of the time	842	61.00
Some of the time	365	26.40
Half of the time	67	4.90
Most of the time	73	5.30
All of the time	34	2.50
Q5/ Poor vision?		
None of the time	886	64.20
Some of the time	308	22.30
Half of the time	57	4.10
Most of the time	78	5.60
All of the time	52	3.80
Have problems with your eyes limited you in performing any of the following during the last week?		
Q6/ Reading?		
None of the time	963	69.70
Some of the time	261	18.90
Half of the time	62	4.50
Most of the time	57	4.10
All of the time	38	2.80
Q7/ Driving at night?		
None of the time	1118	81.00
Some of the time	166	12.00
Half of the time	34	2.50
Most of the time	32	2.30
All of the time	31	2.20
Q8/ Working with a computer or bank machine (ATM)?		
None of the time	927	67.10
Some of the time	312	22.60
Half of the time	64	4.60
Most of the time	57	4.10
All of the time	21	1.50
Q9/ Watching TV?		
None of the time	1055	76.40
Some of the time	213	15.40
Half of the time	49	3.50
Most of the time	42	3.00
All of the time	22	1.60
Have your eyes felt uncomfortable in any of the following situations during the last week?		
Q10/ Windy conditions?		
None of the time	848	61.40
Some of the time	312	22.60
Half of the time	75	5.40
Most of the time	85	6.20
All of the time	61	4.40
Q11/ Places or areas with low humidity (very dry)?		
None of the time	979	70.90
Some of the time	234	16.90
Half of the time	56	4.10
Most of the time	65	4.70
All of the time	47	3.40
Q12/ Areas that are air conditioned?		
None of the time	972	70.40
Some of the time	250	18.10
Half of the time	56	4.10
Most of the time	65	4.70
All of the time	38	2.80
Ocular Surface Disease Index Score (Lowest possible score = 0, highest possible score = 100)		
Minimum	0	
Maximum	48	
Mean	6.65	
Standard deviation	8.1	

**Figure 3:**
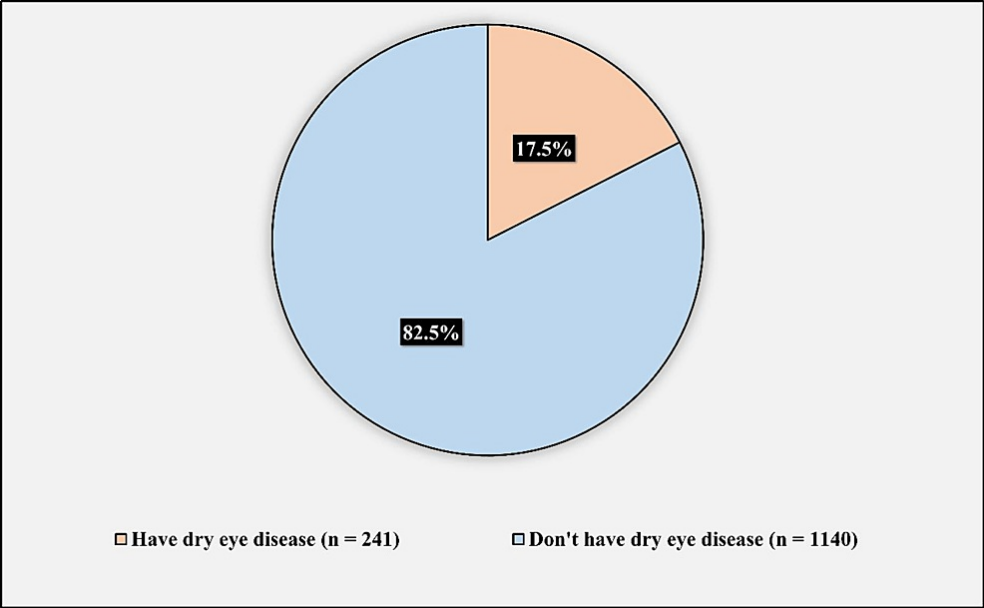
Prevalence of dry eye disease among the participants

Figure 3 shows the prevalence of DED among the participants. The prevalence of DED among the participants was 241 (17.5%), while 1,140 (82.5%) did not have DED.

**Figure 4:**
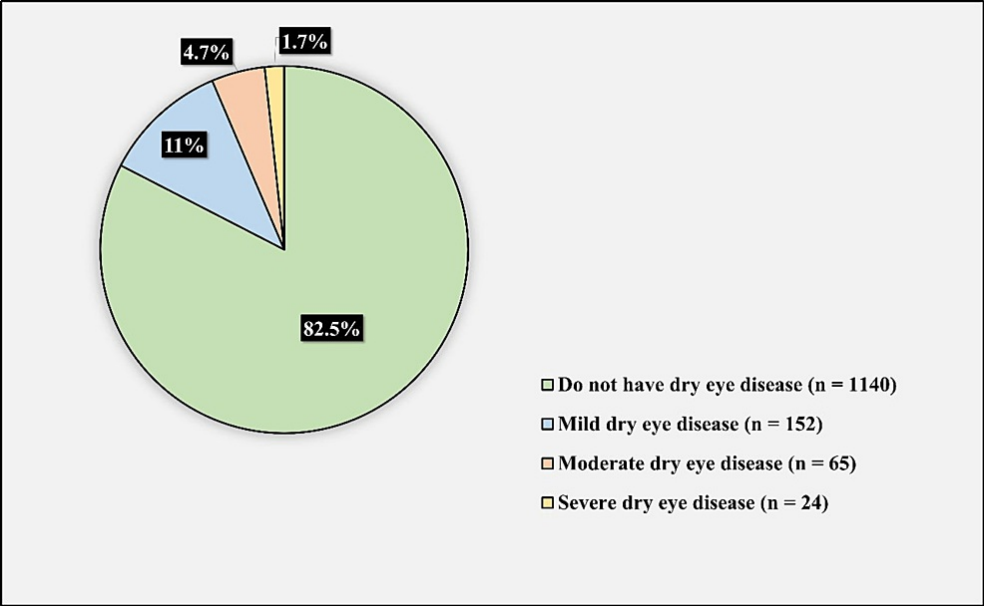
Severity of dry eye disease

Figure 4 displays the severity of DED. 152 (11%) of the participants had mild DED which constitute (63.07%) of those affected with DED, 65 (4.7%) had moderate DED which constitute (26.97%) of those affected with DED, and 24 (1.7%) of the participants had severe DED which constitute (9.96%) of those affected with DED.

**Figure 5:**
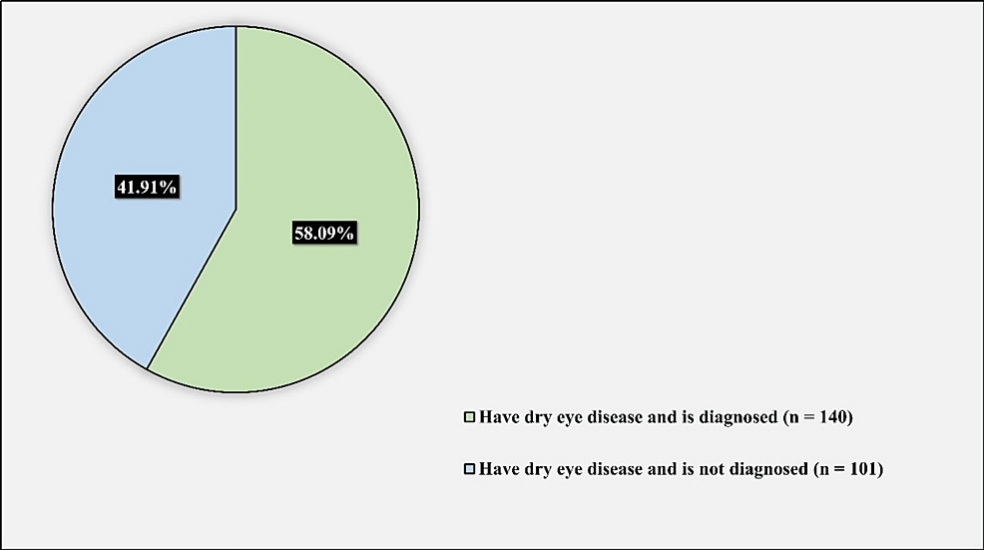
The rate of undiagnosed dry eye disease among participants affected with dry eye disease

Figure 5 demonstrates the rate of undiagnosed DED among participants affected with DED. 140 (58.09%) of the participants affected with DED were previously diagnosed with DED, while 101 (41.91%) of the participants affected with DED were not previously diagnosed with DED.

Table 4 illustrates the factors associated with DED. Gender was significantly associated with having DED (p < 0.001), where it was observed that females had a significantly higher rate of DED compared to males (21.6% vs 10.9%). The following factors were also significantly associated with having DED: having thyroid disease (p = 0.007) (DED rate 28.2% vs 16.7% for those without thyroid disease), having systemic lupus erythematosus/rheumatoid arthritis (p = 0.028) (DED rate 33.3% vs 17.1% for those without systemic lupus erythematosus/rheumatoid arthritis), using antidepressants (p = 0.004) (DED rate 34.1% vs 16.9% for those not using antidepressants), using antihistamine/decongestants (p < 0.001) (DED rate 26.7% vs 15.5% for those not using antihistamine/decongestants), use of electronic devices (being in front of screens) for a prolonged time (p < 0.001) (DED rate 19.6% vs 9.5% for those not using electronic devices for prolonged time), using of contact lenses (p = 0.025) (DED rate 22.1% vs 16.3% for those not using contact lenses), and having a history of eye surgery (p = 0.008) (DED rate 24% vs 16.3% for those without a history of eye surgery). It was also observed that those with a history of conjunctival infections or eyelid infections had a significantly higher rate of DED compared to those without history of conjunctival infections or eyelid infections (p < 0.001) (31.9% vs 11.7%). Moreover, those with a history of corneal abrasions/erosions/ulceration had a significantly higher rate of DED compared to their counterpart (p < 0.001) (30.1% vs 16%).

**Table 4:** Factors associated with dry eye disease *Significant at level 0.05

Factor	Dry Eye Disease		P-Value
	Not affected	Affected	
Age			0.852
18-30 years	627 (83.3%)	126 (16.7%)	
30-45 years	323 (81.8%)	72 (18.2%)	
45-60 years	168 (81.2%)	39 (18.8%)	
61 years and older	22 (84.6%)	4 (15.4%)	
Gender			< 0.001*
Male	474 (89.1%)	58 (10.9%)	
Female	666 (78.4%)	183 (21.6%)	
Do you have diabetes mellitus?			0.104
Yes	79 (76.7%)	24 (23.3%)	
No	1,061 (83%)	217 (17%)	
Do you have Thyroid disease (hyperthyroidism or hypothyroidism)?			0.007*
Yes	61 (71.8%)	24 (28.2%)	
No	1,079 (83.3%)	217 (16.7%)	
Do you have systemic lupus erythematosus / rheumatoid arthritis?			0.028*
Yes	18 (66.7%)	9 (33.3%)	
No	1,122 (82.9%)	232 (17.1%)	
Do you use antidepressants?			0.004*
Yes	27 (65.9%)	14 (34.1%)	
No	1,113 (83.1%)	227 (16.9%)	
Do you use antihistamine / decongestants?			< 0.001*
Yes	176 (73.3%)	64 (26.7%)	
No	964 (84.5%)	177 (15.5%)	
Are you a smoker or do you regularly get exposed to fumes or / smoke?			0.324
Yes	246 (80.7%)	59 (19.3%)	
No	894 (83.1%)	182 (16.9%)	
Use of electronic devices (being in front of screens) for prolonged time			< 0.001*
Yes	874 (80.4%)	213 (19.6%)	
No	266 (90.5%)	28 (9.5%)	
Do you use contact lenses?			0.025*
Yes	212 (77.9%)	60 (22.1%)	
No	928 (83.7%)	181 (16.3%)	
Have you ever had conjunctival infections or eyelid infections?			< 0.001*
Yes	269 (68.1%)	126 (31.9%)	
No	871 (88.3%)	115 (11.7%)	
Do you have cataract?			0.071
Yes	18 (69.2%)	8 (30.8%)	
No	1,122 (82.8%)	233 (17.2%)	
Do you have glaucoma?			0.204
Yes	9 (69.2%)	4 (30.8%)	
No	1,131 (82.7%)	237 (17.3%)	
Have you ever had corneal abrasions / erosions / or ulceration?			< 0.001*
Yes	100 (69.9%)	43 (30.1%)	
No	1,040 (84%)	199 (16%)	
Have you ever had an eye surgery?			0.008*
Yes	152 (76%)	48 (24%)	
No	988 (83.7%)	193 (16.3%)	

Table 5 shows factors associated with undiagnosed DED among participants identified to have DED. Gender was significantly associated with being undiagnosed with DED (p = 0.041), where it was observed that males had a higher rate of being undiagnosed with DED compared to females (53.4% vs 38.3%). Despite the presence of variation in rates of being undiagnosed with DED between age groups, age was not significantly associated with being undiagnosed with DED.

**Table 5:** Factors associated with undiagnosed dry eye disease among participants identified to have dry eye disease *Significant at level 0.05

Factor	Diagnosis Status of Dry Eye Disease		P-Value
	Diagnosed	Not diagnosed	
Age			0.132
18-30 years	65 (51.6%)	61 (48.4%)	
30-45 years	49 (68.1%)	23 (31.9%)	
45-60 years	23 (59%)	16 (41%)	
61 years and older	3 (75%)	1 (25%)	
Gender			0.041*
Male	27 (46.6%)	31 (53.4%)	
Female	113 (61.7%)	70 (38.3%)	

Table 6 displays the multivariate logistic regression (factors predicting the prevalence of DED). The logistic regression model included the following variables: age, gender, having DM, having thyroid disease, having systemic lupus erythematosus, using antidepressants, using antihistamine/decongestants, being a smoker or being exposed to smoke/fumes regularly, using electronic devices (being in front of screens) for a prolonged time, using contact lenses, having cataract, having glaucoma, and having a history of eye surgery. The following factors were observed to be significant risk factors for DED: being a female (p < 0.001, odds ratio = 2.36, 136% increased risk), using antidepressants (p = 0.026, odds ratio = 2.19, 119% increased risk for DED), using antihistamine/decongestants (p = 0.002, odds ratio = 1.71, 71% increased risk for DED), use of electronic devices (being in front of screens) for a prolonged time (p < 0.001, odds ratio = 2.33, 133% increased risk for DED).

**Table 6:** Multivariate logistic regression (factors predicting the prevalence of dry eye disease) * Significant at level 0.05

Factor		P-Value	Odds Ratio	Confidence Interval	
Gender (male vs female)		< 0.001*	2.36	1.64	3.39
Age		0.794	1.00	0.99	1.02
Having diabetes mellitus (yes vs no)		0.274	1.35	0.79	2.30
Having thyroid disease (yes vs no)		0.198	1.42	0.83	2.40
Having systemic lupus erythematosus / rheumatoid arthritis (yes vs no)		0.173	1.85	0.76	4.46
Using antidepressants (yes vs no)		0.026*	2.19	1.10	4.36
Using antihistamine / decongestants (yes vs no)		0.002*	1.71	1.21	2.41
Being a smoker or being regularly exposed to fumes or / smoke (yes vs no)		0.075	1.39	0.97	1.98
Use of electronic devices (being in front of screens) for prolonged time (yes vs no)		< 0.001*	2.33	1.50	3.63
Using contact lenses (yes vs no)		0.951	0.99	0.69	1.42
Having cataract (yes vs no)		0.274	1.70	0.66	4.41
Having glaucoma (yes vs no)		0.469	1.61	0.44	5.85
Had a history of eye surgery (yes vs no)		0.109	1.36	0.93	2.00

## Discussion

As aforementioned, DED is one of the most frequent conditions for patients to consult an ophthalmologist for; as it causes multiple disabling symptoms and as it can affect a person’s functional ability of visualization, like the ability of reading, use a computer, or drive a car at night [1]. Realizing the high potency of disability the DED has, and the lack of nationwide studies that assess the prevalence of DED in Saudi Arabia, it was found essential to study the prevalence of DED and determine the vulnerable groups by assessing the risk factors of DED.

According to McCarty et al. and Uchino et al., the estimated prevalence of DED varies widely and ranges from 5.5% to 73.5% [7,8]. This dramatic variation in the prevalence of DED can possibly be attributed to the lack of standardized diagnostic criteria as well as the controversy about the accuracy of the known diagnostic tests [1,9]. Because of that, the National Eye Institute Workshop has done much research and tests to standardize and unite the way of diagnosing DED. They concluded that not only objective clinical studies of DED are sufficient, but also, an assessment of the subjective symptoms of the disease must be included [6]. Based on the capability of using subjective assessment for screening and diagnosing DED, and due to the aim of this study to target the general population, the validated 12-item questionnaire “Ocular Surface Disease Index” (OSDI) was utilized to estimate the prevalence of DED among residents of Saudi Arabia as it is more applicable to use validated questionnaire to screen for the prevalence of DED, rather than using objective assessment when targeting the general population.

This study revealed that the prevalence of DED among the 1,381 participants was 17.5%. Among those affected with DED, 63.07% had mild DED, 26.97% had moderate DED, and only 9.96% had severe DED. Contrary to this finding, a study conducted in the eastern region of Saudi Arabia among the general population living in AlAhsa city including 1,851 adults has shown that the overall prevalence of having one or more of the six DED symptoms was 32.1% (using the validated six-item questionnaire), which is a much higher prevalence compared to this study [2]. Moreover, a study that was performed in Jeddah, Saudi Arabia on the general population (not having an ocular disease) included 251 participants and revealed a greater rate of DED prevalence of 93.2%. This study tested for DED by using dry eye symptoms questionnaire and an ocular examination by tear film break-up time, fluorescein corneal staining and Schirmer’s test [1]. In a more recent study, a cross-sectional one conducted in Saudi Arabia on 310 adults among the general population determined that DED prevalence was 85%. DED in this study was tested using the OSDI questionnaire and the eye dryness part from contact lens questionnaire-8 (CLDEQ-8) [10]. This high variation of DED prevalence in studies done in Saudi Arabia can be explained by the differences in DED diagnostic criteria that are being used. Where some studies relied seldom on validated questionnaires, and others included objective assessments using physical examination, which can lead to different rates of detecting DED. The sample size of the population and the targeted population could be another explanation for this variation as well, where it is assumed that studies with larger sample sizes are more accurate and reliable.

Globally speaking, the estimated prevalence of DED among the general clinical optometry patient population in Australia is 10.8% which was tested by using the McMonnies dry eye survey in combination with tear film and ocular surface staining [11]. This survey has a similar rate to what this study has reported but is much lower than the other Saudi Arabian studies. Another study done in the United States has reported that the overall prevalence of DED is 6.8% using National Health and Wellness Survey (NHWS) and that 2.7% of the affected participants were from 18 to 34 years of age, and 18.6% of the affected participants were 75 years of age and older, which suggests that there is an increase in prevalence with age [12]. Although the USA study and our study came to an agreement in regard to the relatively low prevalence of DED, our study disagreed with the USA study where there was no significant association between age and the prevalence of DED. Furthermore, a study conducted in Brazil showed that the overall prevalence of DED among Brazilian undergraduate students was found to be 34.39% using the OSDI questionnaire and 23.5% based on the Woman's Health Study questionnaire among the same population [13]. This moreover approves that the prevalence of DED varies extensively depending on the choice of dry eye questionnaire and the objective clinical tests used to diagnose the disease. In addition to that, the characteristics of the targeted subjects of the study might also play a role in the variation of the DED prevalence from one study to another.

This study has found that DED is significantly associated with a variety of factors gender for example. It was found that females were about two times more likely to have DED compared to their male counterparts (21.6% vs 10.9%). Other studies done in the eastern region of Saudi Arabia [2], Ghana [14], United States [12], Ontario, Canada [15], Brazil [13], Nigeria [9] and Palestine [16] reported likewise results that females are more prone to develop DED compared to their male counterparts. Other factors found to be significantly associated with DED in this study were having thyroid disease, having systemic lupus erythematous/rheumatoid arthritis, using antidepressants, antihistamine/decongestants, use of electronic devices, and use of contact lenses. Also, participants with a history of eye surgery, a history of corneal abrasion/erosion/ulceration, and a history of conjunctival or eyelid infections have been observed to have a significantly higher rate of having DED compared to their counterparts. These findings come in agreement with another study carried out in Brazil where they found that contact lens wear, ocular surgery, anti-allergic medications, and rheumatological diseases have a significant association with the prevalence of DED [13]. In the study done in AlAhsa city in the eastern region of Saudi Arabia, DED prevalence was significantly associated with old age and having a history of DM [2]. However, the findings in this study were contrary, there was no correlation found between older age and DED as well as having a history of DM.

In this presented work, multiple factors were observed to be significant risk factors for developing DED. Being a female, using antidepressants, using antihistamine/decongestants, and the use of electronic devices for a prolonged time has been established to be significant predictive factors for developing DED. These findings come in consonance with the study done in Brazil where they found that more than six hours per day of screen time, antidepressant use, and antiallergic medication use are relevant related risk factors for DED. However, in the same study done in Brazil, contact lens use and ocular surgery were also determined as possible predictive factors for developing DED, which was contrary to what was observed in this study [13].

Strengths and limitations

The cross-sectional design of this study and the use of a self-administrative questionnaire may have an impact on the accuracy of the results. Additionally, this study did not combine using objective clinical tests with the subjective questionnaire to diagnose DED and determine its prevalence, which might be considered a pitfall. However, this study provides a useful baseline for the prevalence of DED among the general population of Saudi Arabia. Moreover, although this study's sample size is considered to be high and more than sufficient, a larger sample size from a population-based survey would more accurately reflect the bias-free status of DED prevalence. As nationwide studies cover large areas (such as the entirety of Saudi Arabia) and require as high a sample size as possible. The strength of this study lies in identifying individuals with a high risk of developing DED and spot the light on the necessity to screen them and treat them as early as possible to prevent any further complications and thus increase their quality of life. Another strength point of this presented work is the usage of the validated 12-item questionnaire which was used previously in other nationwide studies.

Recommendation

Although DED has been recognized as one of the commonest ocular complaints in ophthalmology clinics, its diagnosis is still challenging due to the lack of gold-standard methods to reach the diagnosis [17]. For that reason, more studies are needed to identify and standardize the method of diagnosing DED. We also recommend a study that determines which self-administered questionnaire made for diagnosing DED has the highest level of reliability to standardize it as the subjective method for screening for DED. As the study has shown that DED is relatively prevalent in Saudi Arabia, it is recommended to increase awareness of this common condition in society and encourage controlling the modifiable risk factors so DED prevalence and severity can be reduced. There are a variety of ocular surface diseases that might manifest like DED, for that reason, we recommend supporting clinical observation and testing to confirm the diagnosis of DED.

## Conclusions

This study revealed that the prevalence of DED among the general population of Saudi Arabia was 17.5%, which is notably lower than what was observed in other local studies and similar to what was found in some global studies. It is noteworthy to state that a significant majority of those affected had mild DED (63.07%). The usage of antidepressants, antihistamine/decongestant medicines, and having thyroid disorders, systemic lupus erythematosus, and rheumatoid arthritis were all strongly linked to DED. Moreover, using electronics for a prolonged time, using contact lenses, having a history of eye surgery, conjunctivitis or eyelid infections, and having a history of corneal abrasions, erosions, or ulcerations in the past also had a significant association with DED. The significant adjusted risk factors for DED found in this presented work were being a female, using antidepressants, antihistamine/decongestant medicines, and using electronics over a prolonged period of time.

## References

[1] Bukhari A, Ajlan R, Alsaggaf H (2009). Prevalence of dry eye in the normal population in Jeddah, Saudi Arabia. Orbit.

[2] Alshamrani AA, Almousa AS, Almulhim AA (2017). Prevalence and risk factors of dry eye symptoms in a Saudi Arabian population. Middle East Afr J Ophthalmol.

[3] Yasir ZH, Chauhan D, Khandekar R, Souru C, Varghese S (2019). Prevalence and determinants of dry eye disease among 40 years and older population of Riyadh (except capital), Saudi Arabia. Middle East Afr J Ophthalmol.

[4] Song P, Xia W, Wang M (2018). Variations of dry eye disease prevalence by age, sex and geographic characteristics in China: a systematic review and meta-analysis. J Glob Health.

[5] Schein OD, Tielsch JM, Muñoz B (1997). Relation between signs and symptoms of dry eye in the elderly. Ophthalmology.

[6] Schiffman RM, Christianson MD, Jacobsen G, Hirsch JD, Reis BL (2000). Reliability and validity of the Ocular Surface Disease Index. Arch Ophthalmol.

[7] McCarty C (1998). The epidemiology of dry eye in Melbourne, Australia, historical image. Ophthalmology.

[8] Uchino M, Dogru M, Yagi Y (2006). The features of dry eye disease in a Japanese elderly population. Optom Vis Sci.

[9] Betiku AO, Oduyoye OO, Jagun OO, Olajide OS, Adebusoye SO, Aham-Onyebuchi UO (2022). Prevalence and risk factors associated with dry eye disease among adults in a population-based setting in South-West Nigeria. Niger J Clin Pract.

[10] Almutairi AH, Alalawi BS, Badr GH, Alawaz RA, Albarry M, Elbadawy HM (2021). Prevalence of dry eye syndrome in association with the use of contact lenses in Saudi Arabia. BMC Ophthalmol.

[11] Albietz JM (2000). Prevalence of dry eye subtypes in clinical optometry practice. Optom Vis Sci.

[12] Farrand KF, Fridman M, Stillman IÖ, Schaumberg DA (2017). Prevalence of diagnosed dry eye disease in the United States among adults aged 18 years and older. Am J Ophthalmol.

[13] Yang I, Wakamatsu T, Sacho IB (2021). Prevalence and associated risk factors for dry eye disease among Brazilian undergraduate students. PLoS One.

[14] Asiedu K, Kyei S, Boampong F, Ocansey S (2017). Symptomatic dry eye and its associated factors: a study of university undergraduate students in Ghana. Eye Contact Lens.

[15] Caffery B, Srinivasan S, Reaume CJ, Fischer A, Cappadocia D, Siffel C, Chan CC (2019). Prevalence of dry eye disease in Ontario, Canada: a population-based survey. Ocul Surf.

[16] Shanti Y, Shehada R, Bakkar MM, Qaddumi J (2020). Prevalence and associated risk factors of dry eye disease in 16 northern West bank towns in Palestine: a cross-sectional study. BMC Ophthalmol.

[17] Alves M, Reinach PS, Paula JS (2014). Comparison of diagnostic tests in distinct well-defined conditions related to dry eye disease. PLoS One.

